# Bridging the Gap: Human Emotions and Animal Emotions

**DOI:** 10.1007/s42761-022-00125-6

**Published:** 2022-07-16

**Authors:** Michael Mendl, Vikki Neville, Elizabeth S. Paul

**Affiliations:** Animal Welfare and Behaviour Research Group, Bristol Veterinary School, University of Bristol, Bristol, BS40 5DU UK

**Keywords:** Animal emotion, Human emotion, Affective states, Emotion measurement, Translation, Consciousness

## Abstract

Our experiences of the conscious mental states that we call emotions drive our interest in whether such states also exist in other animals. Because linguistic report can be used as a gold standard (albeit indirect) indicator of subjective *emotional feelings* in humans but not other species, how can we investigate animal emotions and what exactly do we mean when we use this term? Linguistic reports of human emotion give rise to *emotion concepts* (discrete emotions; dimensional models), associated objectively measurable behavioral and bodily *emotion indicators*, and understanding of the *emotion contexts* that generate specific states. We argue that many animal studies implicitly translate human emotion *concepts*, *indicators* and *contexts*, but that explicit consideration of the underlying pathways of inference, their theoretical basis, assumptions, and pitfalls, and how they relate to *conscious emotional feelings*, is needed to provide greater clarity and less confusion in the conceptualization and scientific study of animal emotion.

Human emotions are first and foremost *conscious* experiences which we name and categorize using words such as ‘happiness’, ‘pleasure’, ‘fear’, ‘anxiety’, ‘anger’ and ‘sadness’. Our experience of these *feelings*, in all their variations from mild and fleeting to intense and all-consuming, generates and drives our interest in whether non-human animals (hereafter animals) are able to experience something similar and, if so, how this impinges on the quality of their lives. Can rats be depressed (Gururajan et al., 2019)? Can farmed pigs experience boredom or anxiety (Murphy et al., 2014)? Are dogs able to experience envy or guilt (Hecht et al., 2012; Horowitz, 2009, 2012; McGetrick & Range, 2018; Range et al., 2009)? Are insects emotional beings (Anderson & Adolphs, 2014) and, if so, how we should treat them (Mendl et al., 2011)?

Scientific investigations of such questions are important, especially given polarized views ranging from denial of subjective experiences in all other species (e.g., Macphail, 1998) to assertions that, for example, all mammals consciously experience emotions (Panksepp, 1998). However, the challenge we face is that subjective experiences of emotion are inaccessible to direct, objective measurement. In humans, the concept of emotion is largely derived linguistically; language gives us a rich, albeit indirect (and not infallible), insight into people’s conscious emotional experiences (top third of box in Fig. 1). Therefore, self-reported *emotional feelings* are usually taken as the gold-standard measure of emotional states. They also form the basis for higher-level *concepts of emotion* as comprising discrete, modular systems (e.g., a ‘fear’ system) and/or a limited number of underlying dimensions such as valence (positivity / negativity) and arousal (activation) that are common to all emotional states (top two-thirds of box in Fig. 1; Ekman, 1992; Mendl et al., 2010; Mendl & Paul, 2020; Panksepp, 1998; Russell, 2003). Self-reported emotional feelings can also be correlated with accompanying neural, behavioral, physiological and cognitive changes, allowing us to use these as additional *indicators of emotion* in people (Scherer, 1984; bottom two-thirds of box in Fig. 1), and to the situations in which they usually occur, allowing us to identify *emotion-generating contexts* (large arrow above box in Fig. 1).

**Fig. 1:**
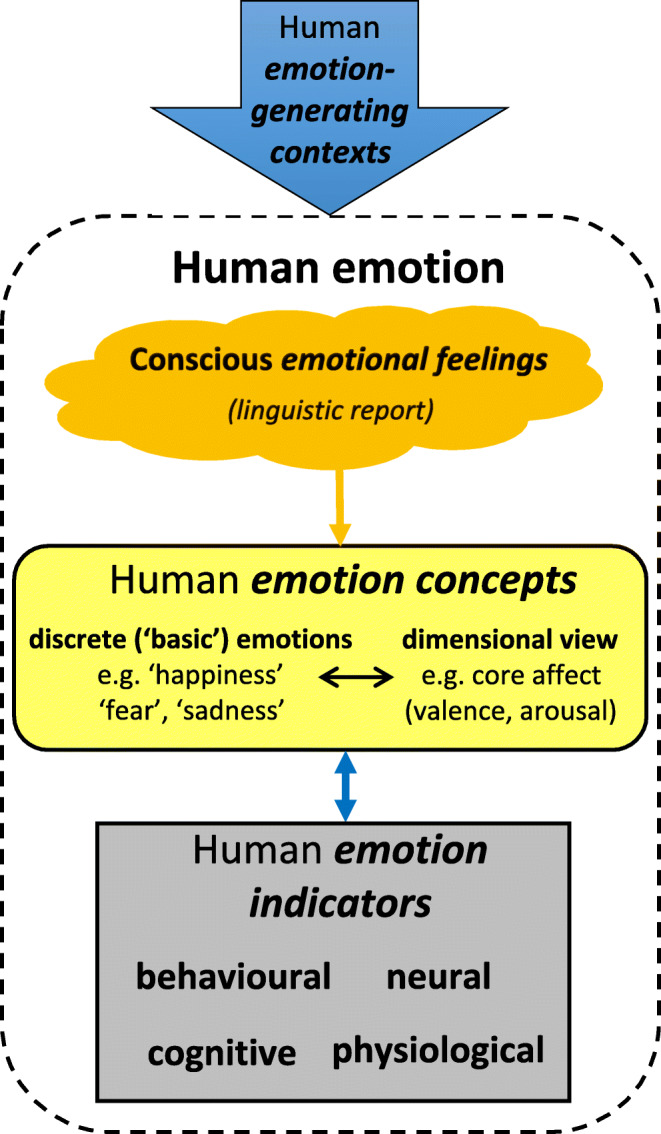
Schematic representation of how linguistic reports of emotional *feelings* are the source of *concepts* of human emotion, including discrete and dimensional models and their associated categories (e.g., ‘fear’, ‘happiness’, ‘core affect’). These categories of reported human feelings can be associated with measurable changes in behavior, physiology, neural and cognitive function which can then be used as additional *indicators* of these states. Likewise, they can be related to the situations in which they usually occur, allowing us to identify emotion-generating *contexts*

In animals, however, we lack linguistic insight into their emotional experiences^1^ and so cannot follow the rationale illustrated in Fig. 1 to identify objectively measurable indicators of emotion. How, therefore, is it possible to study animal emotion scientifically? Figure 2 illustrates pathways of inference that can take us from the phenomena that frame our questions (human emotional feelings) to those that we are able to study in animals (neural, behavioral, physiological, and cognitive responses). We believe that these pathways have been employed by animal emotion researchers for many years, but that this is often done implicitly, generating confusion about what is actually studied and discovered. For example, when researchers report on ‘animal emotion’, ‘fear’, ‘pleasure’ etc., are they referring to conscious feelings and, if not, then what exactly? Scope for confusion becomes even wider when results enter the public domain - headlines may mislead even if article content is more nuanced (e.g., https://www.newscientist.com/article/2107546-dont-worry-bee-happy-bees-found-to-have-emotions-and-moods/). Just as human emotion researchers are still discussing what exactly they mean when they talk about emotion (LeDoux et al., 2016), it is important for animal emotion researchers to be clear too.

**Fig. 2:**
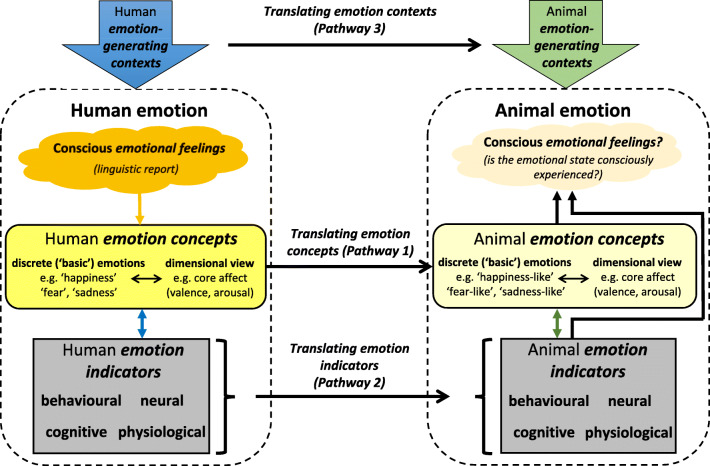
Schematic representation of some of the pathways of inference that are traversed when moving from the source of our interest in animal emotions—human emotional feelings—to what we study in animals—measurable indicators of their emotional states. Concepts of human emotion are ultimately derived from linguistic report as indicated in Fig. 1 and the left-hand side of this figure, and provide the basis for our general concepts of animal emotion (Pathway 1). Behavioral, physiological, neural and cognitive markers that change in reliable ways when people report particular emotional feelings can be used as markers of these states in humans and may also be translated for use as markers of related states in other species (Pathway 2). Reported human feelings are often associated with specific contexts (large ‘emotion-generating contexts’ arrow on left of figure). Likewise, specific contexts can be inferred to induce particular emotional states in animals (Pathway 3). However, whether animal emotional states identified in these ways are consciously experienced (right-hand ‘Conscious emotional feelings?’ bubble) remains the focus of intense research and debate. The assumptions and uncertainties inherent in navigating these pathways are discussed in the text

Here we propose that an explicit awareness of the pathways of inference in Fig. 2, the questions they raise, and their associated assumptions and pitfalls, will help us to identify and avoid some of the confusion surrounding the conceptualization and study of animal emotion. In the following sections, we consider how to navigate these pathways of inference. Having briefly described the links between human emotional feelings, linguistic concepts of human emotion, and indicators and contextual correlates of these emotional states (Fig. 1), we now lay out arguments for and against translating these emotional *concepts* (Pathway 1 in Fig. 2), *indicators* (Pathway 2) and *contexts* (Pathway 3) to other species. We end by briefly considering whether emotional states that we measure in animals are *consciously felt*. Whilst feelings remain inaccessible to direct scientific investigation, we agree with recent papers by de Waal and Andrews (2022) and Kret et al. (2022) that it is unreasonable to assume that only humans consciously experience emotions, and that indirect evidence is accumulating for sentience in a growing range of species. We discuss this in the last section of the paper.

In this article, we use the terms ‘human emotion/affect’ or ‘animal emotion/affect’ to denote the field of study. ‘Emotional states’ are characterised by the defining property of valence (positive/negative; attractive/aversive; rewarding/punishing) and can be either short-lived (emotions) or longer-term (moods). They fall within a broader ‘affect’ or ‘affective state’ category of all valenced-states (e.g., including valenced-components of sensations and motivations); ‘core affect’ being a particular model of affect that comprises valence and arousal dimensions. ‘Emotional states’ and ‘affective states’ have behavioral, neurophysiological, cognitive and subjective (conscious) components. We use the word ‘feelings’ when we refer to the consciously experienced component, and an animal or species capable of experiencing feelings is said to be ‘sentient’. ‘Emotional’ and ‘affective states’ arise in situations that threaten or enhance survival, fitness and achievement of goals, and play a key role in adaptive responses and decision-making. For more information on these points, see also: Carver (2001); de Waal (2011); Ekman and Davison (1994); Mendl et al. (2010); Mendl & Paul (2020); Posner et al. (2005); Russell (2003); Scherer (1984); Watson et al. (1999).

## Translating *Emotion Concepts* from Humans to Animals (Pathway 1): What Types of Emotional State Are Likely to Exist in Other Species?

Here, we highlight issues to consider when following Pathway 1 in Fig. 2 to derive concepts of animal emotion from human emotion research. Animal emotion theorists such as the late Jaak Panksepp (1982; 1998; Panksepp & Watt, 2011) who follow the *discrete* or *basic emotion* approach (Ekman, 1992) argue that certain human emotions (e.g., ‘fear’, ‘anger’) are products of evolutionarily conserved neuro-behavioral systems, and that similar basic emotions are likely to occur in related species (e.g., primates, other mammals). For example, a system that generates fear in people will also exist in other mammals and generate a similar state. This view holds that translating concepts of discrete human emotion to other species is a rational way of framing animal emotion research. Large bodies of work that refer to ‘fear’ and ‘anxiety’ in animals (e.g., Fendt & Fanselow, 1999; Jesuthasan, 2012; Riemer et al., 2021), and the use of other discrete emotion words such as ‘happiness’, ‘sadness’ and ‘depression’ when labelling patterns of behavioral and bodily responses in non-human species, point to the widespread adoption of this approach.

However, when loosely used, these words conjure notions of human-like subjective feelings (LeDoux, 2017) and researchers should clarify whether they mean to imply this. Panksepp himself (1998; see also Panksepp 2010, 2011) identified seven basic (mammalian) emotions: SEEKING, FEAR, RAGE, PANIC, LUST, CARE, PLAY, but was careful to denote them in capital letters to indicate that they were not identical to human feelings. Rather, they referred to brain-based circuits and outputs - ‘natural kinds’ finely adapted for survival and reproduction (see also LeDoux’s 2012 ‘survival circuits’). Clarity on this point is important, and other notations (e.g., ‘anxiety-like’) may be helpful. The use of ‘-like’ terminology risks being interpreted as denying the existence of feelings in other species (cf. de Waal, 1999) or, conversely, tacitly indicating that such feelings do exist and have been validated (Garner, 2014). However, we believe that, when clearly explained, it is a valuable marker of agnosticism about how emotional states studied in animals relate to human (felt) emotions.

Even with this distancing, the claim that animals share discrete emotion systems defined by human emotion words has been subject to accusations of anthropomorphism (e.g., Barrett 2017a,b), and many researchers have considered how this alters with increasing phylogenetic distance, and with the nature of the emotion under consideration. For example, ‘fear-like’ states may be widespread whilst ‘envy-like’ states may be confined to humans and perhaps other mammals (Brosnan & de Waal, 2003; McGetrick & Range, 2018). Moreover, the fact that different theories propose different numbers of human discrete emotions (Ekman, 1992; Izard, 2007; Plutchik, 2001), and that cultural differences exist in human emotion words (e.g., the state of looking worse after a haircut has a specific word in Japanese; ‘age-otori’), challenges the notion of universal discrete emotions in humans, let alone in other species.

In contrast to the discrete emotion approach, proponents of *dimensional models* and theories of *constructed emotion* posit that emotional feelings are infinitely varied. Barrett (2017a) argues that human emotions are dependent on individual conceptualizations of current sensory (interoceptive) input, and hence are strongly shaped by individual life experiences; there are no basic emotion neurobehavioral systems to be conserved across taxa and there is no basis for simple translation of discrete emotion categories, because such categories are essentially human constructions. According to this view, emotion-like states in other species may be shaped by their own sensory and perceptual worlds, and their capacities to construct emotion-like concepts, and hence be very different to those that humans experience (Bliss-Moreau, 2017). Nevertheless, core affect (subjectively experienced valence and arousal states) is seen as one of the fundamental ingredients from which emotional events are constructed (Barrett, 2017b). Given the likely link between core affect and the essential survival activities of acquiring reward, avoiding punishment, and making appropriate decisions, it is highly plausible that core affect systems of some sort exist in many other species (Mendl & Paul, 2020). In this view, ‘positively valenced affect’ or ‘high arousal negative states’ are translatable (Barrett, 2017b).

To summarize, these issues call for researchers to provide clear definitions and rationales when arguing that we can validly translate a particular human discrete emotion concept or a human dimensional model to another species. In particular, explanations are needed of what we mean when using human emotion words in the animal context, and considerations of the biological functions of affects and emotions (e.g., to obtain food or escape predation) are key. We should also remember that if we translate from human emotional concepts, we risk missing out on emotional states that other species may have but we don't. Here, a constructionist approach that emphasises the role that species’ sensory and perceptual worlds play in generating emotional states (Bliss-Moreau, 2017) provides a starting point for thinking about the nature of non-human emotion.

## Translating *Emotion Indicators* from Humans to Animals (Pathway 2): How Can We Assess Animal Emotional States?

Irrespective of which concept of animal emotion we employ, the next challenge is to identify indicators of emotional states that can be measured objectively. Inference Pathway 2 in Fig. 2 involves the translation of emotion indicators identified in humans, to animals. If we are interested in a particular human emotion concept (e.g., ‘happiness’) and want to measure it in animals (‘happiness-like’), we can posit that behavioral and bodily changes occurring in humans reporting ‘happiness’ (and not occurring when happiness is not reported) are valid indicators of this state, and hence measure equivalent states in our study species. Such an argument assumes: (1) that there is close and consistent correspondence between self-reported human emotions and their associated behavioral, physiological, neural, and cognitive profiles; (2) that this relationship is conserved in the animal species concerned.

The possibility that human experiences of *discrete emotions* such as fear, anger, and happiness can be reliably associated with distinctive profiles of bodily and behavioral change has been a topic of research and debate for many years (e.g., Barrett, 2006; Ekman et al., 1983; Kreibig, 2010; Levenson, 1992; Mauss & Robinson, 2009). Consistent clusters of behavioral, physiological, and neural changes have been associated with certain discrete emotions (e.g., Kragel & Labar, 2016; Kreibig, 2010; Vytal & Hamann, 2010), and some behavioral correlates show particular promise. For example, facial expressions have been associated with specific discrete emotions in humans (e.g., gaping mouth and ‘disgust’; Berridge & Winkielman, 2003), and this may also be the case in rodents and a range of other mammalian species (Berridge, 2000). Indeed, there is a growing body of work investigating facial expressions of discrete emotions across a variety of mammals (Caeiro et al., 2013; Dolensek et al., 2020; Langford et al., 2010; McLennan et al., 2019; Parr et al., 2007; Viscardi et al., 2017; Waller et al., 2012).

In contrast, other studies have identified links between the *affective dimension* of valence, rather than discrete emotions, and human physiology and behavior (Lindquist et al., 2012; Mauss & Robinson, 2009). These findings support the constructionist account that experience of discrete emotion is not tied to specific somatic or neural profiles, but that core affect states may be (Barrett, 2006, 2017a,b; Lindquist et al., 2012). There is also evidence that, in humans, the dimension of affective valence can be reliably linked to lateralization of sensory-affective processing (Davidson, 1992) and to cognitive biases in judgements of ambiguity (Mathews & MacLeod, 1994; Paul et al., 2005) raising the possibility that this may also be the case in animals (Mendl et al., 2009, 2010; Siniscalchi et al., 2010). For example, the judgement bias test, which measures ‘optimistic’ or ‘pessimistic’ responses to ambiguous stimuli in animals (Harding et al., 2004; Lagisz et al., 2020; Mendl et al., 2009; Neville et al., 2020), was based on the finding that humans reporting negative affective states tend to make pessimistic judgements whilst those reporting positive states respond optimistically (Muris & van der Heiden, 2006; Paul et al., 2005).

Ongoing research should continue to evaluate the validity and reliability of human indicators of discrete and/or dimensional affective states, and hence their value for use in other species. However, with increasing phylogenetic distance, and associated dissimilarities in behavior, physiology, neurobiology, and ecology, there will be decreasing likelihood that correlations between reported emotional states and particular behavioral and bodily changes observed in humans also hold in other species. Thus, this form of inference will become less plausible as one moves further away from humans on the phylogenetic tree.

A sub-category of this inference pathway concerns our tendency to intuitively interpret human-like behavior patterns in other species as indicators of particular states. For example, it is difficult to avoid assuming that playing mammals are experiencing ‘joy-like’ states (Ahloy-Dallaire et al., 2016; Held & Spinka, 2011; Spinka et al., 2001). Static, hunched postures in captive animals, on the other hand, resemble illness or ‘depression-like’ states, and startle behavior can look very much like ‘fear’. Because these are informal inferences, it is difficult to ascertain their validity and there is a significant risk of error, especially if our knowledge of the species is limited. Even when we know a species well, we can make mistakes (e.g., primate grimacing is often mistaken for ‘happy’ smiling van Hooff, 1976). Furthermore, we will be much less ready to attribute specific affective states to those taxa (e.g., insects) who are phylogenetically distant and whose behavior is less human-like.

If we acknowledge our anthropocentric perspective and apply critical anthropomorphism (Burghardt, 2007; de Waal, 1999), these intuitive perspectives may allow us to carefully identify indicators which can then be studied experimentally. Moreover, some theoretical approaches argue that our ability to intuitively read the behavior of other animals provides us with reliable evidence about their emotional states. Wemelsfelder (1997), for example, has argued from a Rylean philosophical perspective (Ryle, 1949) that animal subjectivity and hence emotional feelings are accessible to direct observation, and that they can be revealed by the expressive quality of behavior (Wemelsfelder, 2001). Her research program has developed qualitative methods to measure the expressive nature of an animal’s ongoing behavior as an indicator of affect (Wemelsfelder et al., 2001). A different theoretical perspective is offered by Anderson and Adolphs (2014) who argue that humans and animals share adaptive ‘emotion primitives’ (scalability, valence, persistence, generalization), and that indicators which exhibit these properties can be considered to be markers of animal emotion.

In summary, researchers should provide clear arguments, reasoning and empirical evidence to support inferences that changes in behavior, physiology, neural activity, or cognition in human subjects reporting particular emotional states can also be used as markers of corresponding affective states in animals. However, these types of inference depend on homology with humans and are therefore most likely to be valuable for closely related species such as primates and other mammals.

## Translating Human *Emotion-Generating Contexts* to Animals (Pathway 3): Can We Establish an Animal’s ‘Ground Truth’ Emotional State at Any One Time and Use This to Identify Indicators of Animal Emotion?

Inference Pathway 3 in Fig. 2 stems from the recognition that events, situations, and contextual characteristics are crucial players in the induction of human emotions (‘Human emotion-generating context’ arrow in Fig. 2). Translation of this principle to animals (‘Animal emotion-generating context’ arrow in Fig. 2) involves inferring that particular contexts induce particular emotional states in animals too.^2^ For example, many humans find that being alone in an unfamiliar, dark place at night can be fear-inducing, presumably because such situations threatened the survival of our ancestors. If, therefore, we place a burrow-dwelling nocturnal rat in a brightly-lit open field, we can infer that this context will, likewise, induce fear- or anxiety-like states. The accompanying changes to that rat’s behavior and physiology can then be identified as representative measures of the rodent’s affective state. There are, however, potential problems with this approach. Our anthropocentric perspective means that we may fail to detect contextual influences that influence animal affect, including smells and sounds in the case of the rat (Burn, 2008).

The use of theoretical frameworks and/or operational definitions of animal affect and emotion may help to formalize context-based inference by spelling out underlying assumptions. For example, it is possible to formalize the largely intuitive process outlined in the previous paragraph by theorizing that any threats to an animal’s survival and/or fitness can be assumed to produce negative affective states, while contexts that improve fitness can be assumed to produce positive states (Burgdorf & Panksepp, 2006). Of course, the veracity of inferred links between context and affective valence will depend on the quality of our knowledge about the species’ biology, ecology, and life-history strategy. And mistakes may be made; animals may not always accurately detect fitness threats or benefits, they may encounter conflicts (e.g., when reproduction and death co-occur as in some invertebrate species Andrade 1996; Maxwell, 1998), and there may be evolved reasons for animals to experience some potential fitness hazards as rewarding (e.g., predator inspection Godin & Davis, 1995).

Some of these problems can be avoided by employing operational definitions of animal affect that specify the relationship between context and emotion. For example, one such definition (Gray 1987; Millenson, 1967; Paul & Mendl, 2018; Rolls, 2005) grounded in reinforcement theories of affective valence states that…

Animal affective states are elicited by rewards and punishers or their predictors. A reward is anything for which an animal will work, and a punisher is anything that it will work to escape or avoid. Rewards or the absence of punishers, and associated predictions thereof, induce positive affect. Punishers or the absence of rewards, and associated predictions thereof, induce negative affect…. (Mendl & Paul, 2020, building on Rolls, 2005, 2014)

This definition, operationalized by defining rewards and punishers in terms of the behavior of animals that encounter them, allows researchers to assume that when an animal is exposed to a context containing reward (something that it will work to acquire) it will be in a positively-valenced affective state, and when exposed to a punisher (something it will work to avoid) it will be in a negatively-valenced affective state. In other words, the researcher identifies the ‘ground-truth’ affective state of the animal by reference to a specified context, and any changes in behavior, physiology, and neural markers that occur reliably can be taken as indicators of positive or negative affect. At a practical level, establishing ‘ground-truth’ - the state that the animal is actually in - is a major challenge when attempting to develop indicators of animal affect, and failure to do this effectively may underlie many inconsistencies in studies that seek to validate new measures (cf. Lagisz et al., 2020; Neville et al., 2020). The operational definition outlined here, which can be applied across taxa, can thus help to address this problem. At the very least, it clearly states the assumptions underlying inferences about the links between contexts and affective valence.

The translation of appraisal theories from human emotion research to animals provides an example of context-based inference which applies to discrete emotions. Appraisal theories propose that the way in which different contextual features are appraised generates the felt emotional state (Ellsworth, 2013; Moors et al., 2013; Panksepp, 2007). For example, people appraising a stimulus or situation as *sudden* and low in *familiarity*, *predictability*, *pleasantness* and *consistency with expectations,* tend to report feeling ‘fear’, whilst those appraising a stimulus as moderately *predictable*, *highly pleasant*, *consistent with expectations* and *not sudden* tend to report ‘happiness’ (Sander et al., 2005). Researchers have thus proposed that contexts that occur suddenly and are low in familiarity, predictability, pleasantness, and consistency with expectations can be inferred to induce a state of ‘fear’ in animals too, and that the resulting profile of behavioral, physiological and neural changes provide markers of ‘fear’ in that animal or species (Désiré et al., 2004; Veissier et al., 2009). For example, Désiré et al. (2006) induced differing states in sheep by manipulating the suddenness and familiarity of stimuli; sudden onset of a stimulus generated startle and an increase in heart rate, while an unfamiliar stimulus generated a behavioral orienting response. Such approaches depend, of course, on the extent to which emotion-specific appraisal characteristics observed in humans can be translated to other species and, once more, are likely to be less plausible in more distantly related taxa.

To summarize, whilst simple translation of human-focused emotive contexts to animals is risky, it is possible to formalize such processes by grounding them in structured theoretical frameworks, including reinforcement theory and appraisal theory. Whilst both can be questioned in terms of their underlying assumptions (Paul & Mendl, 2018), their strengths are evident in providing transparent, objective, repeatable, and empirically tractable ways of inferring animal emotional states, important steps forward from intuitive inference.

## Is the Measured Emotional State Consciously Experienced?

By employing the approaches discussed above, supported by rigorous argument for the pathways of inference shown in Fig. 2, it is possible to systematically measure animal affective states, laying out clear arguments for why the measures taken reflect particular discrete emotions, or affective valence and arousal. However, to conclude that corresponding conscious emotional *feelings* exist in the study species remains problematic (‘Conscious emotional feelings?’ bubble in Fig. 2).

Knowing the conscious emotional experiences of humans is not straightforward given the fallibility of linguistic report, the range of societal and cultural influences that impinge on emotional concepts, and the challenge of assessing feelings in non-linguistic infants and others. However, knowing about conscious emotions in other species is even more challenging. Returning to Panksepp’s seven basic emotions, he was clear that they are accompanied by some sort of subjective experience, at least in mammalian species, but that this may well be qualitatively different to human emotional feelings; conscious emotional experience does occur in other mammals, but the nature of this experience is unknowable (Panksepp, 1998, 2010, 2011). LeDoux (2012), on the other hand, placed further distance between the study of animal affective states and that of human emotional feelings, advocating the use of emotion neutral terminology such as ‘defensive survival circuits’ rather than ‘fear’, because human emotion words are too easily interpreted as implying the existence of subjective feelings in other species. For LeDoux, inferring conscious emotional experience from animal emotion indicators is not justifiable (LeDoux, 2012; LeDoux & Brown, 2017).

Whether researchers are prepared to take this final leap of inference about feelings depends largely upon acceptance of certain philosophical arguments (e.g., that animal subjectivity is evident in the expressive quality of behavior Wemelsfelder (1997) and/or proposed lines of evidence for a species’ capacity to consciously experience mental states including emotions (Panksepp, 2005). There is no shortage of debate on this topic, including arguments about whether fish can feel pain (Braithwaite, 2010; Key, 2016) and whether insects are conscious (Barron & Klein, 2016). There are theoretical perspectives on the neural underpinnings of conscious emotion in non-humans, and on the behavioral and neural signs that we might use to identify the capacity for consciousness (Boly et al., 2013; LeDoux & Brown, 2017; Panksepp, 2010; Paul et al., 2020; Rolls, 2005; Seth et al., 2005). There are also articles claiming that consciousness is widespread (Bekoff, 2006), or denying consciousness in any species apart from humans (Macphail, 1998). A consensus is yet to be established, but a 2012 position paper (Low et al., 2012; Cambridge Declaration on Consciousness) argues that there is now sufficient weight of evidence to support the existence of neural substrates that generate consciousness in mammals, birds, and some other species such as octopus.

Further advances in studies of animal consciousness will be needed to traverse this last inference pathway (Paul et al., 2020). Demonstrations of behavioral and neural processes paralleling those observed in humans reporting specific conscious feelings are particularly compelling. For example, recent studies provide behavioral and neural evidence for a ‘regret-like’ state in rats following decisions revealed to be economically-costly by counter-factual information. These include orbitofrontal cortex activation also observed in humans reporting regret (Steiner & Redish, 2014).

## Conclusion

In this paper, we have described and discussed the pathways of inference that take us from an understanding of human emotion to one of animal emotion. We have identified key steps in the reasoning process that researchers use, often implicitly, to frame the study of animal emotion, including the translation of *emotion concepts*, *emotion indicators*, and *emotion contexts* from humans to animals. We believe that awareness of these steps will help pinpoint some of the assumptions underlying our inferences, their associated uncertainties, and the need for explicit and clear arguments to decrease confusion and misunderstanding in this rapidly expanding field. While human *emotion concepts* vary from theory to theory, it is likely that processes akin to human affective dimensions (of valence and arousal), and perhaps some discrete-emotion like states, may be translatable to non-human animals. Inferring emotion-like states in animals may also be possible, both through theory-dependent identification of *emotion-generating contexts*, and through homology-based identification of *emotion indicators* (e.g., behavioral and physiological markers of valence, arousal, etc.). However, to complete the final transition from our own experience of *emotional feelings* (essentially the driver of public concern and scientific interest in animal emotion) to the measurement of conscious feelings in other species remains a major challenge, but exciting progress is being made. Meeting this challenge is important because animal sentience - the capacity to consciously experience affective states - is widely recognized in ethics and law as underpinning our animal welfare obligations. A better understanding of sentience in other species will therefore have significant implications for how we should treat animals in our care.

## Footnotes

1 Arguably, a form of linguistic self-report may be achievable in certain species (e.g., great apes taught sign language Krause & Beran, 2020; parrots Pepperberg, 2021), but the problem of determining when a learnt symbol - object/action/state association actually reflects a full ‘understanding’ of the symbolic reference is not trivial (Pepperberg, 2021).

2 Here we concentrate on contexts *external* to the subject but, in humans at least, ‘internal’ thoughts, reflections, future plans, simulations, and other cognitions also generate emotions.

## References

[CR1] Ahloy-Dallaire J, Espinosa J, Mason G (2016). Play and optimal welfare: Does play indicate the presence of positive affective states?. Behavioural Processes.

[CR2] Anderson DJ, Adolphs R (2014). A framework for studying emotions across species. Cell.

[CR3] Andrade MC (1996). Sexual selection for male sacrifice in the Australian redback spider. Science.

[CR4] Barrett LF (2006). Are emotions natural kinds?. Perspectives on Psychological Science.

[CR5] Barrett, L. F. (2017a). *How Emotions Are Made: The Secret Life of the Brain.* Houghton Mifflin Harcourt.

[CR6] Barrett LF (2017). The theory of constructed emotion: an active inference account of interoception and categorization. Social Cognitive and Affective Neuroscience.

[CR7] Barron AB, Klein C (2016). What insects can tell us about the origins of consciousness. Proceedings of the National Academy of Sciences of the United States of America.

[CR8] Bekoff M (2006). The public lives of animals. Journal of Consciousness Studies.

[CR9] Berridge KC, Winkielman P (2003). What is an unconscious emotion? (The case for unconscious “liking”). Cognition and Emotion.

[CR10] Berridge KC (2000). Measuring hedonic impact in animals and infants: Microstructure of affective taste reactivity patterns. Neuroscience and Biobehavioral Reviews.

[CR11] Bliss-Moreau E (2017). Constructing nonhuman animal emotion. Current Opinion in Psychology.

[CR12] Boly M, Seth AK, Wilke M, Ingmundson P, Baars B, Laureys S, Edelman DB, Tsuchiya N (2013). Consciousness in humans and non-human animals: Recent advances and future directions. Frontiers in Psychology.

[CR13] Braithwaite V (2010). *Do Fish Feel Pain?*.

[CR14] Brosnan SF, de Waal FBM (2003). Monkeys reject unequal pay. Nature.

[CR15] Burgdorf J, Panksepp J (2006). The neurobiology of positive emotions. Neuroscience and Biobehavioral Reviews.

[CR16] Burghardt GM (2007). Critical anthropomorphism, uncritical anthropocentrism and naïve nominalism. Comparative Cognition and Behaviour Reviews.

[CR17] Burn CC (2008). What is it like to be a rat? Rat sensory perception and its implications for experimental design and rat welfare. Applied Animal Behaviour Science.

[CR18] Caeiro CC, Waller BM, Zimmermann E, Burrows AM, Davila-Ross M (2013). OrangFACS: A muscle-based facial movement coding system for orangutans (Pongo spp.). International Journal of Primatology.

[CR19] Carver CS (2001). Affect and the functional bases of behavior: On the dimensional structure of affective experience. Personality and Social Psychology Review.

[CR20] Davidson RJ (1992). Anterior cerebral asymmetry and the nature of emotion. Brain & Cognition.

[CR21] de Waal FBM (1999). Anthropomorphism and anthropodenial: consistency in our thinking about humans and other animals. Philosophical Topics.

[CR22] de Waal FBM (2011). What is an animal emotion?. Annals of the New York Academy of Sciences.

[CR23] de Waal FBM, Andrews K (2022). The question of animal emotions. Science.

[CR24] Désiré L, Veissier I, Despres G, Boissy A (2004). On the way to assess emotions in animals: Do lambs (Ovis aries) evaluate an event through its suddenness, novelty, or unpredictability?. Journal of Comparative Psychology.

[CR25] Désiré L, Veissier I, Despres G, Delval E, Toporenko G, Boissy A (2006). Appraisal process in sheep (Ovis aries): Interactive effect of suddenness and unfamiliarity on cardiac and behavioral responses. Journal of Comparative Psychology.

[CR26] Dolensek N, Gehrlach DA, Klein AS, Gogolla N (2020). Facial expressions of emotion states and their neuronal correlates in mice. Science.

[CR27] Ekman P (1992). An argument for basic emotions. Cognition and Emotion.

[CR28] Ekman P, Davison RJ (1994). *The Nature of Emotion*.

[CR29] Ekman P, Levenson RW, Friesen WV (1983). Autonomic nervous system activity distinguishes among emotions. Science.

[CR30] Ellsworth PC (2013). Appraisal theory: Old and new questions. Emotion Review.

[CR31] Fendt M, Fanselow M (1999). The neuroanatomical and neurochemical basis of conditioned fear. Neuroscience and Biobehavioral Reviews.

[CR32] Garner J (2014). The significance of meaning: why do over 90% of behavioural neuroscience results fail to translate to humans, and what can we do to fix it?. ILAR Journal.

[CR33] Gray JA (1987). *The Psychology of Fear and Stress*.

[CR34] Godin JGJ, Davis SA (1995). Who dares, benefits: predator approach behaviour in the guppy (Poecilia reticulata) deters predator pursuit. Proceedings of the Royal Society of London. Series B: Biological Sciences.

[CR35] Gururajan A, Reif A, Cryan JF, Slattery DA (2019). The future of rodent models in depression research. Nature Reviews Neuroscience.

[CR36] Harding EJ, Paul ES, Mendl M (2004). Animal behaviour - cognitive bias and affective state. Nature.

[CR37] Hecht J, Miklosi A, Gacsi M (2012). Behavioral assessment and owner perceptions of behaviors associated with guilt in dogs. Applied Animal Behaviour Science.

[CR38] Held SDE, Spinka M (2011). Animal play and animal welfare. Animal Behaviour.

[CR39] Horowitz A (2009). Disambiguating the "guilty look": Salient prompts to a familiar dog behaviour. Behavioural Processes.

[CR40] Horowitz A (2012). Fair is fine, but more is better: limits to inequity aversion in the domestic dog. Social Justice Research.

[CR41] Izard CE (2007). Basic emotions, natural kinds, emotion schemas, and a new paradigm. Perspectives on Psychological Science.

[CR42] Jesuthasan S (2012). Fear, anxiety, and control in the zebrafish. Developmental Neurobiology.

[CR43] Key B (2016). Why fish do not feel pain. Animal Sentience.

[CR44] Kragel PA, Labar KS (2016). Decoding the nature of emotion in the brain. Trends in Cognitive Sciences.

[CR45] Krause MA, Beran MJ (2020). Words matter: reflections on language projects with chimpanzees and their implications. American Journal of Primatology.

[CR46] Kreibig SD (2010). Autonomic nervous system activity in emotion: A review. Biological Psychology.

[CR47] Kret ME, Massen JJM, de Waal FBM (2022). My fear is not, and never will be, your fear: on emotions and feelings in animals. Affective Science.

[CR48] Lagisz M, Zidar J, Nakagawa S, Neville V, Sorato E, Paul ES, Bateson M, Mendl M, Lovlie H (2020). Optimism, pessimism and judgement bias in animals: A systematic review and meta-analysis. Neuroscience and Biobehavioral Reviews.

[CR49] Langford DJ, Bailey AL, Chanda ML, Clarke SE, Drummond TE, Echols S, Glick S, Ingrao J, Klassen-Ross T, Lacroix-Fralish ML, Matsumiya L, Sorge RE, Sotocinal SG, Tabaka JM, Wong D, van den Maagdenberg A, Ferrari MD, Craig KD, Mogil JS (2010). Coding of facial expressions of pain in the laboratory mouse. Nature Methods.

[CR50] LeDoux JE (2012). Rethinking the emotional brain. Neuron.

[CR51] LeDoux JE (2017). Semantics, surplus meaning, and the science of fear. Trends in Cognitive Sciences.

[CR52] LeDoux JE, Brown R (2017). A higher-order theory of consciousness. Proceedings of the National Academy of Sciences of the United States of America.

[CR53] LeDoux J, Phelps L, Alberini C (2016). What we talk about when we talk about emotions. Cell.

[CR54] Levenson RW (1992). Autonomic nervous system differences among emotions. Psychological Science.

[CR55] Lindquist KA, Wager TD, Kober H, Bliss-Moreau E, Barrett LF (2012). The brain basis of emotion: A meta-analytic review. Behavioral and Brain Sciences.

[CR56] Low, P., Panksepp, J., Diana Reiss, D., Edelman, D., Van Swinderen, B. & Koch, C. (2012). *Cambridge Declaration on Consciousness*. Publicly proclaimed at the *Francis Crick Memorial Conference on Consciousness in Human and non-Human Animals* at Churchill College, University of Cambridge. https://fcmconference.org/img/CambridgeDeclarationOnConsciousness.pdf

[CR57] Macphail EM (1998). *The Evolution of Consciousness*.

[CR58] Mauss IB, Robinson MD (2009). Measures of emotion: A review. Cognition & Emotion.

[CR59] Mathews A, MacLeod C (1994). Cognitive approaches to emotion and emotional disorders. Annual Review of Psychology.

[CR60] Maxwell MR (1998). Lifetime mating opportunities and male mating behaviour in sexually cannibalistic praying mantids. Animal Behaviour.

[CR61] McGetrick J, Range F (2018). Inequity aversion in dogs: a review. Learning & Behavior.

[CR62] McLennan KM, Miller AL, Dalla Costa E, Stucke D, Corke MJ, Broom DM, Leach MC (2019). Conceptual and methodological issues relating to pain assessment in mammals: The development and utilisation of pain facial expression scales. Applied Animal Behaviour Science.

[CR63] Mendl, M., Burman, O. H. P., & Paul, E. S. (2010). An integrative and functional framework for the study of animal emotion and mood. *Proceedings of the Royal Society B-Biological Sciences*, (*277*), 2895–2904. 10.1098/rspb.2010.0303 PMC2982018 20685706

[CR64] Mendl M, Burman OHP, Parker RMA, Paul ES (2009). Cognitive bias as an indicator of animal emotion and welfare: Emerging evidence and underlying mechanisms. Applied Animal Behaviour Science.

[CR65] Mendl M, Paul ES (2020). Animal affect and decision-making. Neuroscience and Biobehavioral Reviews.

[CR66] Mendl MT, Paul ES, Chittka L (2011). Animal behaviour: Emotion in invertebrates?. Current Biology.

[CR67] Millenson, J. R. (1967). *Principles of Behavioral Analysis.* Macmillan Company.

[CR68] Moors A, Ellsworth PC, Scherer KR, Frijda NH (2013). Appraisal theories of emotion: State of the art and future development. Emotion Review.

[CR69] Muris P, Van der Heiden S (2006). Anxiety, depression, and judgments about the probability of future negative and positive events in children. Journal of Anxiety Disorders.

[CR70] Murphy E, Nordquist RE, van der Staay FJ (2014). A review of behavioural methods to study emotion and mood in pigs, *Sus scrofa*. Applied Animal Behaviour Science.

[CR71] Neville V, Nakagawa S, Zidar J, Paul ES, Lagisz M, Bateson M, Lovlie H, Mendl M (2020). Pharmacological manipulations of judgement bias: A systematic review and meta-analysis. Neuroscience and Biobehavioral Reviews.

[CR72] Panksepp J (1982). Toward a general psycho-biological theory of emotions. Behavioral and Brain Sciences.

[CR73] Panksepp, J. (1998). *Affective Neuroscience: The Foundations of Human and Animal Emotions.* Oxford University Press.

[CR74] Panksepp J (2005). Affective consciousness: core emotional feelings in animals and humans. Consciousness and Cognition.

[CR75] Panksepp J (2007). Neurologizing the psychology of affects: how appraisal-based constructivism and basic emotion theory can co-exist. Perspectives on Psychological Science.

[CR76] Panksepp J (2010). Affective consciousness in animals: perspectives on dimensional and primary process emotion approaches. Proceedings of the Royal Society B – Biological Sciences.

[CR77] Panksepp J (2011). The basic emotional circuits of mammalian brains: Do animals have affective lives?. Neuroscience and Biobehavioral Reviews.

[CR78] Panksepp J, Watt D (2011). What is basic about basic emotions? lasting lessons from affective neuroscience. Emotion Review.

[CR79] Parr LA, Waller BM, Vick SJ, Bard KA (2007). Classifying chimpanzee facial expressions using muscle action. Emotion.

[CR80] Paul ES, Mendl MT (2018). Animal emotion: Descriptive and prescriptive definitions and their implications for a comparative perspective. Applied Animal Behaviour Science.

[CR81] Paul ES, Harding EJ, Mendl M (2005). Measuring emotional processes in animals: The utility of a cognitive approach. Neuroscience and Biobehavioral Reviews.

[CR82] Paul ES, Sher S, Tamietto P, Winkielman P, Mendl MT (2020). Towards a comparative science of emotion: Affect and consciousness in humans and animals. Neuroscience and Biobehavioral Reviews.

[CR83] Pepperberg IM (2021). Nonhuman and nonhuman-human communication: Some issues and questions. Frontiers in Psychology.

[CR84] Plutchik R (2001). The nature of emotions - human emotions have deep evolutionary roots, a fact that may explain their complexity and provide tools for clinical practice. American Scientist.

[CR85] Posner J, Russell JA, Peterson BS (2005). The circumplex model of affect: an integrative approach to affective neuroscience, cognitive development, and psychopathology. Development and Psychopathology.

[CR86] Range F, Horn L, Viranyi Z, Huber L (2009). The absence of reward induces inequity aversion in dogs. Proceedings of the National Academy of Sciences of the United States of America.

[CR87] Riemer S, Heritier C, Windschnurer I, Pratsch L, Arhant C, Affenzeller N (2021). A review on mitigating fear and aggression in dogs and cats in a veterinary setting. Animals.

[CR88] Rolls ET (2005). *Emotion Explained*.

[CR89] Rolls ET (2014). *Emotion and Decision Making Explained*.

[CR90] Ryle, G. (1949). *The Concept of Mind.* Hutchinson.

[CR91] Russell JA (2003). Core affect and the psychological construction of emotion. Psychological Review.

[CR92] Sander D, Grandjean D, Scherer KR (2005). A systems approach to appraisal mechanisms in emotion. Neural Networks.

[CR93] Scherer KR, Scherer KR, Ekman P (1984). On the nature and function of emotion: A component process approach. *Approaches to Emotion*.

[CR94] Seth AK, Baars BJ, Edelman DB (2005). Criteria for consciousness in humans and other mammals. Consciousness and Cognition.

[CR95] Siniscalchi M, Sasso R, Pepe AM, Vallortigara G, Quaranta A (2010). Dogs turn left to emotional stimuli. Behavioural Brain Research.

[CR96] Spinka M, Newberry RC, Bekoff M (2001). Mammalian play: Training for the unexpected. Quarterly Review of Biology.

[CR97] Steiner AP, Redish AD (2014). Behavioral and neurophysiological correlates of regret in rat decision-making on a neuroeconomic task. Nature Neuroscience.

[CR98] van Hoof JARAM, Bruner JS, Jolly A, Sylva K (1976). A comparative approach to the phylogeny of laughter and smiling. *Play, Its Role In Development And Evolution*.

[CR99] Veissier I, Boissy A, Désiré L, Greiveldinger L (2009). Animals' emotions: studies in sheep using appraisal theories. Animal Welfare.

[CR100] Viscardi AV, Hunniford M, Lawlis P, Leach M, Turner PV (2017). Development of a piglet grimace scale to evaluate piglet pain using facial expressions following castration and tail docking: A pilot study. Frontiers in Veterinary Science.

[CR101] Vytal K, Hamann S (2010). Neuroimaging support for discrete neural correlates of basic emotions: A voxel-based meta-analysis. Journal of Cognitive Neuroscience.

[CR102] Waller BM, Lembeck M, Kuchenbuch P, Burrows AM, Liebal K (2012). GibbonFACS: A muscle-based facial movement coding system for hylobatids. International Journal of Primatology.

[CR103] Watson D, Wiese D, Vaidya J, Tellegen A (1999). The two general activation systems of affect: structural findings, evolutionary considerations, and psychobiological evidence. Journal of Personality and Social Psychology.

[CR104] Wemelsfelder F (1997). The scientific validity of subjective concepts in models of animal welfare. Applied Animal Behaviour Science.

[CR105] Wemelsfelder F (2001). The inside and outside aspects of consciousness: complementary approaches to the study of animal emotion. Animal Welfare.

[CR106] Wemelsfelder F, Hunter TEA, Mendl M, Lawrence AB (2001). Assessing the ‘whole animal’: a free choice profiling approach. Animal Behaviour.

